# Supplementary material for: Sorting at embryonic boundaries requires high heterotypic interfacial tension
Source: Nat Commun. 2017 Jul 31;8:157. doi: 10.1038/s41467-017-00146-x (PMC5537356; doi:10.1038/s41467-017-00146-x)
Supplement: Supplementary file 2 — Supplementary Software 1 [file 41467_2017_146_MOESM2_ESM.zip › PottsModel/SrcPottsModel/doc/model/IEnergyFunction.html]

IEnergyFunction


JavaScript is disabled on your browser.


Skip navigation links


- Overview
- Package
- Class
- Use
- Tree
- Deprecated
- Index
- Help

- Prev Class
- Next Class

- Frames
- No Frames

- All Classes

- Summary:
- Nested |
- Field |
- Constr |
- Method

- Detail:
- Field |
- Constr |
- Method


model

## Interface IEnergyFunction

- ---

    

  ```
  public interface IEnergyFunction
  ```

- - ### Method Summary

    All Methods Instance Methods Abstract Methods

    | Modifier and Type | Method and Description |
    | --- | --- |
    | `double` | `computeEnergyLocally(Lattice aLattice, Pixel pSource, Pixel pTarget)` Computes the energy of the two pixels and their neighbours. |
    | `double` | `computeEnergyLocallyIfSwapped(Lattice aLattice, Pixel pSource, Pixel pTarget)` |
    | `double` | `getLocalPixelInteractions(Pixel pPixel)` Computes the boundary energy around a single pixel. |

- - ### Method Detail


    - #### computeEnergyLocally

      ```
      double computeEnergyLocally(Lattice aLattice,
                                  Pixel pSource,
                                  Pixel pTarget)
      ```

      Computes the energy of the two pixels and their neighbours. These are the
      only pixel energies that change when pPixel1 and pPixel2 are swapped,
      thus they are useful when recomputing the total energy of the lattice.

      Parameters:
      :   `aLattice` -
      :   `pSource` -
      :   `pTarget` -

      Returns:


    - #### computeEnergyLocallyIfSwapped

      ```
      double computeEnergyLocallyIfSwapped(Lattice aLattice,
                                           Pixel pSource,
                                           Pixel pTarget)
      ```


    - #### getLocalPixelInteractions

      ```
      double getLocalPixelInteractions(Pixel pPixel)
      ```

      Computes the boundary energy around a single pixel. It corresponds to
      interaction energy between the given pixel and all its neighbours as
      defined in getAllOrthogonalNeighbours().

      Returns:
      :   the boundary energy surrounding the specified pixel. This is the
          sum of 4 individual energies.


Skip navigation links


- Overview
- Package
- Class
- Use
- Tree
- Deprecated
- Index
- Help

- Prev Class
- Next Class

- Frames
- No Frames

- All Classes

- Summary:
- Nested |
- Field |
- Constr |
- Method

- Detail:
- Field |
- Constr |
- Method
